# Towards a better understanding of the role of stabilizers in QESD crystallizations

**DOI:** 10.1007/s11095-022-03212-2

**Published:** 2022-03-09

**Authors:** Jerome Hansen, Peter Kleinebudde

**Affiliations:** Institute of Pharmaceutics and Biopharmaceutics, Heinrich Heine Universitaet Duesseldorf, Universitaetsstrasse 1, 40225 Duesseldorf, Germany

**Keywords:** morphology, particle engineering, polymeric stabilizers, spherical crystallization, viscosity

## Abstract

Quasi-emulsion solvent-diffusion crystallization (QESD) is a type of spherical crystallization which can be used as a particle design method to improve the flowability and micromeritic properties of drugs or excipients. Spherical particles are generated by dispersing a solvent phase in an antisolvent so that a transient emulsion is formed. Within the droplets the material can crystallize and agglomerate into spherical, hollow particles. Surfactants, such as surface-active polymers like hypromellose, are often required to stabilize the quasi-emulsion. To gain further understanding for the role of the stabilizer, a new screening-method was developed which compared different surface active polymers in solution at similar dynamic viscosities rather than at a set concentration. The dynamic viscosities of a low-viscosity grade hypromellose solution used in the previous publications describing the QESD crystallization of metformin hydrochloride by the authors was used as a target value. QESD crystallizations of metformin hydrochloride (MF) and celecoxib showed that the type of stabilizer and whether it is dissolved in the solvent or antisolvent has an effect on the agglomerates. For MF, the type of hypromellose used can have a significant influence on the properties of the agglomerates. More polymers could be used to stabilize the transient emulsion of celecoxib than previously found in literature. Furthermore, QESD crystallizations seem to be more robust when the stabilizer is dissolved in the antisolvent, however this can lead to a reduced drug load of the agglomerates.

**Supplementary Information:**

The online version contains supplementary material available at 10.1007/s11095-022-03212-2.

## Introduction

Good flowability of powders can be essential for further processing, to ensure, for example, a homogenous blending process and reproducible filling of the die during tableting. Different product attributes, such as residual moisture (1, 2), particle size distribution (3–5), particle morphology (4, 5) and electrostatic charging (6, 7) can affect the flowability of powders. Many drugs form needles or other poorly flowing crystal structures, so that further processing, like granulation or spray-drying, or the addition of glidants is required to produce a free-flowing powder.

As the crystallization of an API or excipient is often part of the final production step, using a crystallization method which can favorably change the micromeritic properties of the product is advantageous. During typical crystallizations, such as cooling, evaporation or antisolvent addition, particle size and morphology can be influenced by, e.g., the super saturation (8), agitation rate (9) or choice of solvent (10).

The quasi-emulsion solvent-diffusion (QESD) crystallization method is a type of spherical crystallization alongside the spherical agglomeration and ammonia-diffusion method. QESD crystallizations are typically based on antisolvent crystallizations where surfactants are used to create a transient emulsion of the solute solution within the antisolvent. The counter-diffusion of the solvents into and out of the quasi-emulsion droplets leads to an increase of the saturation within, which eventually leads to the formation of a crust and spherical, hollow agglomerates. Studies have shown that drugs crystallized by this method can not only have improved flowability (11), but also an improved tabletability (12, 13), reduced punch-sticking (14), storage agglomeration (11) and improved dissolution rates (15).

The transient emulsion formed during QESD crystallizations has been stabilized using different surfactants, such as cellulose-derivatives (11, 14, 16), non-ionic surfactants (17, 18) and poloxamers (19). Some studies have shown that for certain APIs/excipients (20–22) no surfactants are needed. So far, most of the drugs crystallized via the QESD method have been poorly soluble in water, which means, that the polymers were dissolved in the aqueous, antisolvent phase. In previous studies published by the authors (11, 13), a QESD crystallization method for metformin hydrochloride (MF), a highly water-soluble drug, has been described. Hypromellose (HPMC), Pharmacoat® 603 in particular, was used as a stabilizer in the aqueous, solvent phase. Pharmacoat® 603 is a 2910-substitution type HPMC with a low nominal viscosity. Other publications using HPMC as a QESD stabilizer have used the 2208-type (14, 23–25) and a comparison of the two has not yet been published.

As only a few QESD publications show the screening process used by the authors (14, 26) with a limited variety of surfactants, the authors wanted to show this on a broader scale. The aim was to gain further insights into the role of the stabilizer. The authors hoped to see whether the surfactants are only required to stabilize the transient emulsion through an increase in viscosity and a reduction in the interfacial tension, or whether specific interactions between the solute and stabilizer are of greater importance. Furthermore, an improved screening-method is presented where the concentration of the polymers is adjusted so that the solutions have a similar dynamic viscosity, as changes in viscosities can have an influence on agglomerate properties (16). Screening methods which compare polymers at a constant concentration are seen as problematic by the authors, as different polymers form solutions with different viscosities.

A further aim of this study was to see whether the substitution-type and molecular weight of HPMC can have an influence on the micromeritic properties of the MF agglomerates and if other surface-active polymers could be used as well. Lastly, the authors also wanted to see, whether the rules developed for MF could be transferred to a poorly water soluble API, celecoxib, as the polymers are dissolved in the antisolvent, rather than the solute solution for these systems.

## Materials and methods

### Materials

The QESD crystallizations of metformin hydrochloride (MF, Auro Laboratories Ltd., India), pramipexole dihydrochloride (Chr. Olesen, Denmark), selegilin hydrochloride (BASF, Germany), metoprolol tartrate (Microsin, Romania) and salbutamol sulfate (Caelo, Germany) were performed by dissolving the API in demineralized water and crystallizing in technical-grade acetone. Drugs with a poor solubility in water, celecoxib (CEL, Cadila Pharmaceuticals Limited, India) and naproxen sodium (NP, Divi’s Laboratories Limited, India) were dissolved in technical-grade acetone and crystallized in demineralized water. A variety of pharmaceutical grade polymers and surfactants (Table I) were added to the aqueous phase. For this screening hydroxypropylmethylcellulose (HPMC), hydroxypropylcellulose (HPC), hydroxyethylcellulose (HEC), polyvinylpyrrolidone (PVP), polyvinylpyrrolidone/vinyl acetate copolymer (PVPVA), polyvinyl alcohol-polyethylene glycol graft copolymer (PVA-PEG) and polysorbate 80 (PS80) were used (Table I).

**Table I:** Polymers used as QESD-stabilizers

Stabilizer	Type & Name		Labeled Viscosity / mPa∙s		Manufacturer
HPMC	Substitution type 2910	Pharmacoat® 603	2 % (w/w)	3	Shin-Etsu Chemical Co., Ltd., Japan
		Pharmacoat® 645	2 % (w/w)	4.5	
		Pharmacoat® 606	2 % (w/w)	6	
		Pharmacoat® 615	2 % (w/w)	15	
		Metolose® 60SH-50	2 % (w/w)	50	
		Methocel™ E4M	2 % (w/w)	4000	Caelo, Germany
	Substitution type 2208	Methocel™ K4M	2 % (w/w)	4000	Colorcon, UK
HPC	SSL SFP		2 % (w/w)	2.0-2.9	Nippon Soda Co., Ltd., Japan
	L		2 % (w/w)	6.0-10.0	
	M		2 % (w/w)	150-400	
HEC	Natrosol™ 250 G Pharm		2 % (w/w)	250-400	Ashland Specialty Ingredients, USA
PVP	Kollidon® 90 F		10 % (v/w)	300-700	BASF SE, Germany
PVPVA	Kollidon® VA 64		K-value	64	BASF SE, Germany
PVA-PEG	Kollicoat® IR		20 % (w/w)	115	BASF SE, Germany
PS80	Tween 80		-	-	Caelo, Germany

### QESD crystallization of metformin hydrochloride

As described in the previous publications (11, 13), the crystallizations of MF were performed in a 1000 mL jacketed, glass crystallizer (ID = 80 mm) with four-bladed PTFE-coated propeller (50 mm diameter) connected to an overhead stirrer (Eurostar 60 control, IKA, Germany) set to 250 rpm. The crystallizer was temperature controlled using a circulating thermostat (DD-200F, Julabo, Germany). The antisolvent was set to 20 °C at the start of the crystallization. The aqueous solutions of MF were prepared according to Table II, where the respective stabilizer was added to the aqueous solvent phase. The stabilizer was firstly fully dissolved in water at room temperature. 40 % (w/w) MF was then added to the solution and heated to 50 ± 1 °C to ensure full dissolution of the drug. For each trial 35.5 mL MF solution was crystallized in 600 g acetone. The MF solutions were pumped into the crystallizer through 0.75 mm ID ETFE tubing using a syringe pump (Legato 100, KD Scientific, USA) equipped with a jacketed 50 mL glass syringe (SGE Analytical Science, Australia) kept at 50 °C using a secondary circulating thermostat (BC4, Julabo, Germany). Once the entire solution was added, the system was stirred for an additional 30 min. Filtration, washing with pure antisolvent (acetone) and drying at 60 °C of the agglomerates was performed on 63 μm stainless steel sieves.

**Table II:** Concentrations of stabilizers in the aqueous solution for CEL crystallizations and the resulting concentration when 40 % (w/w) MF was added to the solution

Polymer	Type & Name		Concentration / % (w/w)	Resulting conc. with 40 % MF / % (w/w)
HPMC	Substitution Type 2910	Pharmacoat® 603	2.44	1.46
		Pharmacoat® 645	1.80	1.08
		Pharmacoat® 606	1.48	0.89
		Pharmacoat® 615	0.87	0.52
		Metolose® 60SH-50	0.55	0.33
		Methocel™ E4M	0.18	0.11
	Substitution Type 2208	Methocel™ K4M	0.21	0.13
HPC	SSL SFP		3.32	1.99
	L		1.26	0.76
	M		0.31	0.19
HEC	Natrosol™ 250 G Pharm		0.32	0.19
PVP	Kollidon® 90 F		1.25	0.75
PVPVA	Kollidon® VA 64		7.50	4.50
PVA-PEG	Kollicoat® IR		5.00	3.00
PS80	Tween 80		0.0080	0.0048

### QESD crystallization of celecoxib

Due to the limited availability of the material the experiments had to be conducted at a smaller scale than those of MF. The crystallizations were performed in a 150 mL glass beaker using a magnetic stirrer set to 500 rpm. Similar to the QESD crystallization techniques already described in literature (14, 26, 27), 3 g CEL was dissolved in 9 mL technical-grade acetone at room temperature and crystallized in the aqueous polymer solutions (Table II). For each trial, 10 mL CEL solution was added to 100 g antisolvent using a syringe pump set to 1.0 mL/min using 0.75 mm ID ETFE tubing. Once the entire solution was added, the system was stirred for an additional 30 min. Filtration, washing with pure antisolvent (demineralized water) and drying at 60 °C of the agglomerated was performed on 63 μm stainless steel sieves. To verify that the QESD crystallization of CEL could also be performed on a larger scale, two crystallizations were performed using the apparatus and volumes used for MF.

### Dynamic viscosity

The dynamic viscosity of the aqueous polymer solutions (1 mL) without API was determined using a rotational rheometer (Kinexus pro, Malvern Instruments, UK) equipped with a cone (1°/Ø 60 mm) and plate geometry (Ø 65 mm). Measurements with API were not possible, as MF was not sufficiently soluble at room temperature and evaporation at higher temperatures caused precipitation of the drug. The dynamic viscosities were determined by applying a constant shear stress of 1 Pa while heating the samples from 20 to 70 °C at a rate of 7.5 °C/min. Measurements were taken every 5 s. Each solution was measured in triplicate.

### Surface tension

The surface tension of the aqueous polymer solutions without API (approximately 30 mL) was measured at 25 °C using an automatic force tensiometer K100 (Krüss, Germany) equipped with a Wilhelmy plate which was freshly annealed before each measurement. The samples were equilibrated for 5 min before each measurement to ensure a constant temperature. Per measurement, 10 values were recorded in 5 s intervals. The measurements were performed in triplicate.

### Particle size distribution (PSD)

The PSDs of the MF agglomerates were determined using dynamic image analysis (Haver CPA 2-1, Haver & Boecker OHG, Germany) equipped with an ultrasonic dispersion unit. In this device, the agglomerates fall between an LED-light source and a line scan camera which is used to determine the PSD. The entire batch was measured each time.

### Microscopy

A light microscope (Leica DMLB, Leica Camera AG, Germany) equipped with a camera was used to obtain microscopic images of the agglomerates. Scanning electron microscopy (SEM) images were obtained using a Phenom G2 pro (Phenom-World BV, Netherlands) without sputtering at an accelerating voltage of 5-10 kV under vacuum. The samples were secured using conductive carbon tape.

### Powder flow

The bulk and tapped densities of the HPMC-MF agglomerates were determined using the apparatus described by the European Pharmacopoeia 10.0 (2.9.34.; n=3) (28) using a 250 mL graduated cylinder. The Hausner Ratio (HR) was calculated using the bulk volume (BV) and tapped volume (TV) after 1250 taps (HR = BV/TV; HR ≤ 1.18 = “good” flowability).

The angle of repose was determined according to the European Pharmacopoeia 10.0 (2.9.36.; n=3) (29) using an 8 cm in diameter base plate. A powder has a “good” flowability if the angle of repose is ≤ 35°.

### Differential Scanning Calorimetry (DSC)

DSC measurements were conducted using a 1 STARe system (Mettler-Toledo, Germany). The samples (ca. 3 mg, n=2) were heated from 20 to 260 °C at a rate of 10 K/min in sealed aluminum pans.

### Drug content

The drug content of the MF agglomerates crystallized in the different HPMC types and PVPVA was determined using UV/Vis-measurements (UV-1800 SHIMADZU, Japan) at 233 nm using a 10 mm quartz cuvette. For this, 125 mg of the MF agglomerates, weighed exactly, were dissolved in 500 mL demineralized water and then diluted at a ratio of 1:25. The measurements were repeated six-fold.

## Results and discussion

The QESD crystallization technique relies on the formation of transient emulsion droplets of the API solution within the antisolvent so that spherical agglomerates can be formed. The stabilization of an emulsion can be achieved by, e.g., increasing the viscosity of one or more phases and/or by reducing the interfacial tension of the system (30, 31).

In literature, a variety of polymers and surfactants have been used for the QESD crystallization of different APIs, however no clear rules have been developed as to their exact role in stabilizing the transient emulsion. On the one hand, the role could be to simply stabilize the emulsion droplets as the described stabilizers can increase the viscosity and/or reduce the interfacial tension of the system (32). This allows for a slower counter-diffusion of solvent and antisolvent (33). On the other hand, these polymers can interact with the solute and therefore reduce the nucleation rate (34, 35). Furthermore, they can adsorb onto the crystal faces at the interface, slow down the crystal growth (34, 36) and thereby generate a smooth agglomerate surface. Studies have shown that a strong interaction between the API and polymer can be required for the formation of spherical agglomerates (14, 26).

Typically, when polymer screenings for QESD are described, QESD crystallizations are performed using different polymers at a constant concentration and the resulting agglomerates are characterized (14, 26). This is seen as critical by the authors as the polymers can differ in their nominal viscosities, as these depend on, e.g., the type of polymer and its molecular weight. Therefore, a screening technique using a fixed concentration, e.g. 0.1 % w/w, of the different polymers can result in solutions with different viscosities. This can lead to differences in the droplet sizes of the quasi-emulsion formed during the crystallization process (32). Furthermore, Maghsoodi *et al*. (16) showed that if the viscosity of the antisolvent solution is too high, by adding too much HPC, spherical agglomerates can no longer be produced. Therefore, a screening technique comparing polymers and surfactants in constant weight-percentage can be misleading because the failure to form spherical agglomerates might not be due to an insufficient interaction of the polymer and the API but rather that the viscosity of the system was simply too high. For this study it was therefore proposed, to adjust the concentrations of the used polymers so that the resulting solutions have similar dynamic viscosities. By doing so, the droplet size of the API solution should be similar throughout the different experiments, so that the influence of the polymers can be better observed.

**Fig. 1:**
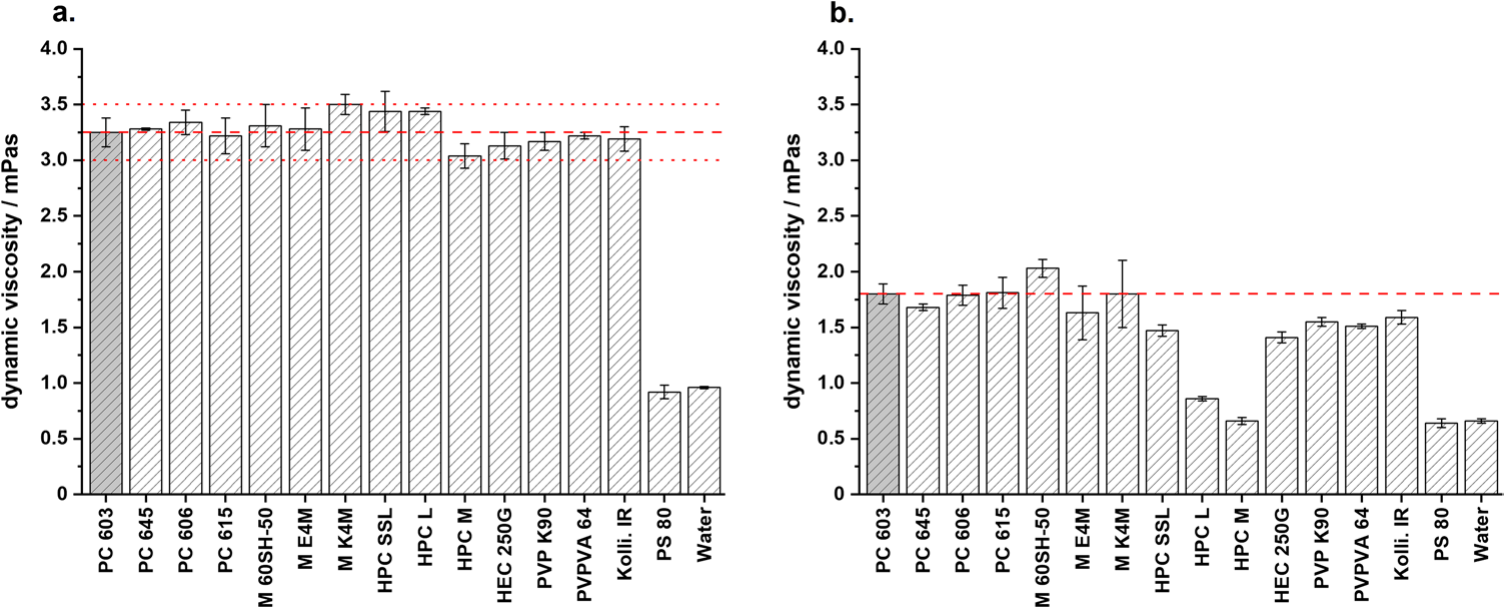
Dynamic viscosities of the aqueous polymer solutions without API at **a.** 25 °C and **b.** 50 °C (mean ± s, n=3)

The original crystallization technique developed by the authors for MF used a low-viscosity 2910-HPMC type, Pharmacoat® 603 in particular, as a stabilizer for the transient emulsion. In this study, the authors set out so see whether the molecular weight and substitution type of HPMC has an influence on the agglomerate properties. Furthermore, seven additional polymers were evaluated for their suitability. The required concentrations were determined through a trial-and-error method, so that all aqueous solutions had a similar dynamic viscosity to the 2.44 % (w/w) Pharmacoat® 603 solution (3.25 mPa•s ± 0.13) used in the original formulation (Fig. 1a). A deviation of 0.25 mPa•s was tolerated; the concentrations can be seen in Table II. Polysorbate 80 was also used as the non-ionic surfactant only reduced the interfacial tension of the aqueous solution to that of the PC 603 one without greatly changing the viscosity of the solution.

**Fig. 2:**
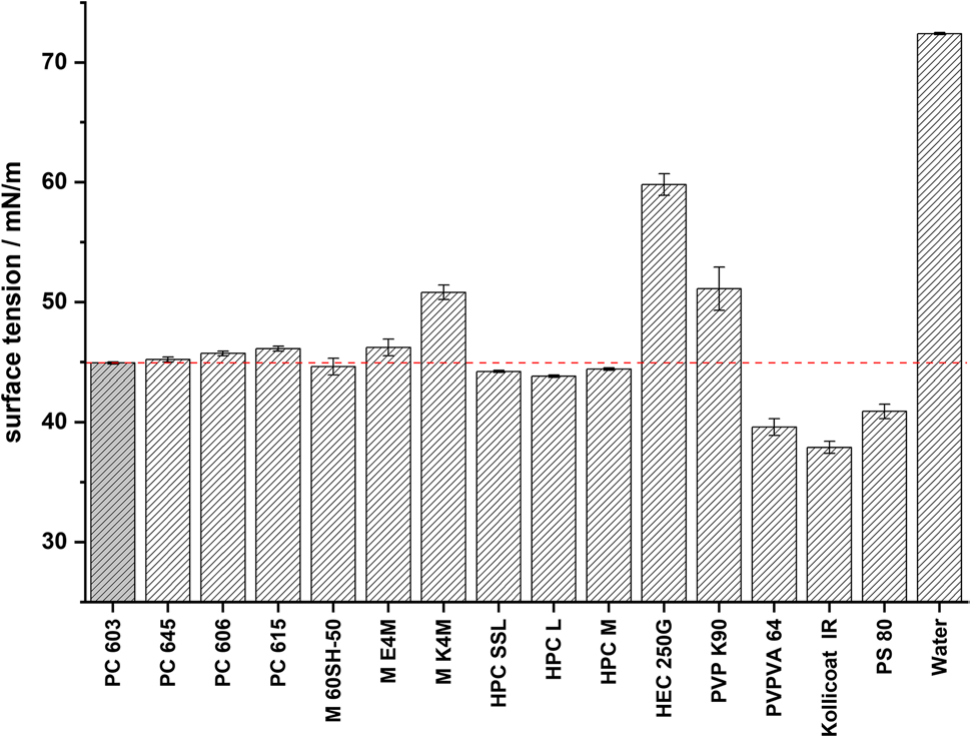
Surface tension of the aqueous polymer solutions without API (mean ± s, n=3)

Since the stability of an emulsion is also dependent on the interfacial tension present between the two phases, the surface tension of the aqueous solutions was measured (Fig. 2). It was interesting to observe, that most of the solutions had a similar surface tension compared to that of the PC 603 solution (44.9 ± 0.1 mN/m), even though the concentrations varied up to a factor of 10. Only HPMC K4M, which differs in its substitution type from the others HPMCs analyzed, HEC, PVP K90, Kollidon® VA 64 and Kollicoat® IR deviated from the line. Since the viscosities and the surface tension of the aqueous polymer solutions were similar, it can be expected that the droplet size should therefore be similar during these QESD crystallizations.

### Influence of the HPMC type

So far in literature, an evaluation of different HPMC grades and substitution-types as stabilizers for QESD crystallizations has not been done. In general, a reduction in the amount of stabilizer required would be preferential, as material costs can be reduced and the drug load of the agglomerates can increase with less stabilizer used (16). However, this has to be evaluated for each product specifically, as adsorbed polymer can have a positive effect on the product qualities, such as a reduction in punch-sticking during tableting (14) or reduced storage agglomeration (11).

As the aqueous MF solutions containing the polymer were heated to 50 °C to achieve full dissolution of the API, the dynamic viscosities of the aqueous solutions were measured at 50 °C as well (Fig. 1b). Overall, the dynamic viscosities of these solutions were lower than at 25 °C, however all HPMC types remained above their respective cloud-point.

When comparing the properties of the MF agglomerates produced with 2910-HPMC types with different molecular weights, a definite change in the agglomerate properties could be observed. A reduction in the concentration of HPMC in the MF solutions with increasing molecular weight, while keeping all other variables (such as viscosity, stirrer rpm, solution feed-rate, temperature, etc.), did not greatly affect the PSD of the agglomerates. Only those crystallized in the presence of PC 606 and 615 slightly deviated to smaller PSDs (Fig. 4a). This shift could be confirmed by a repetition of the experiments. Measurements of the PSD generally showed that the droplet size of the quasi-emulsion and the resulting PSD of the agglomerates can be controlled by the viscosity of the solution.

**Fig. 3:**
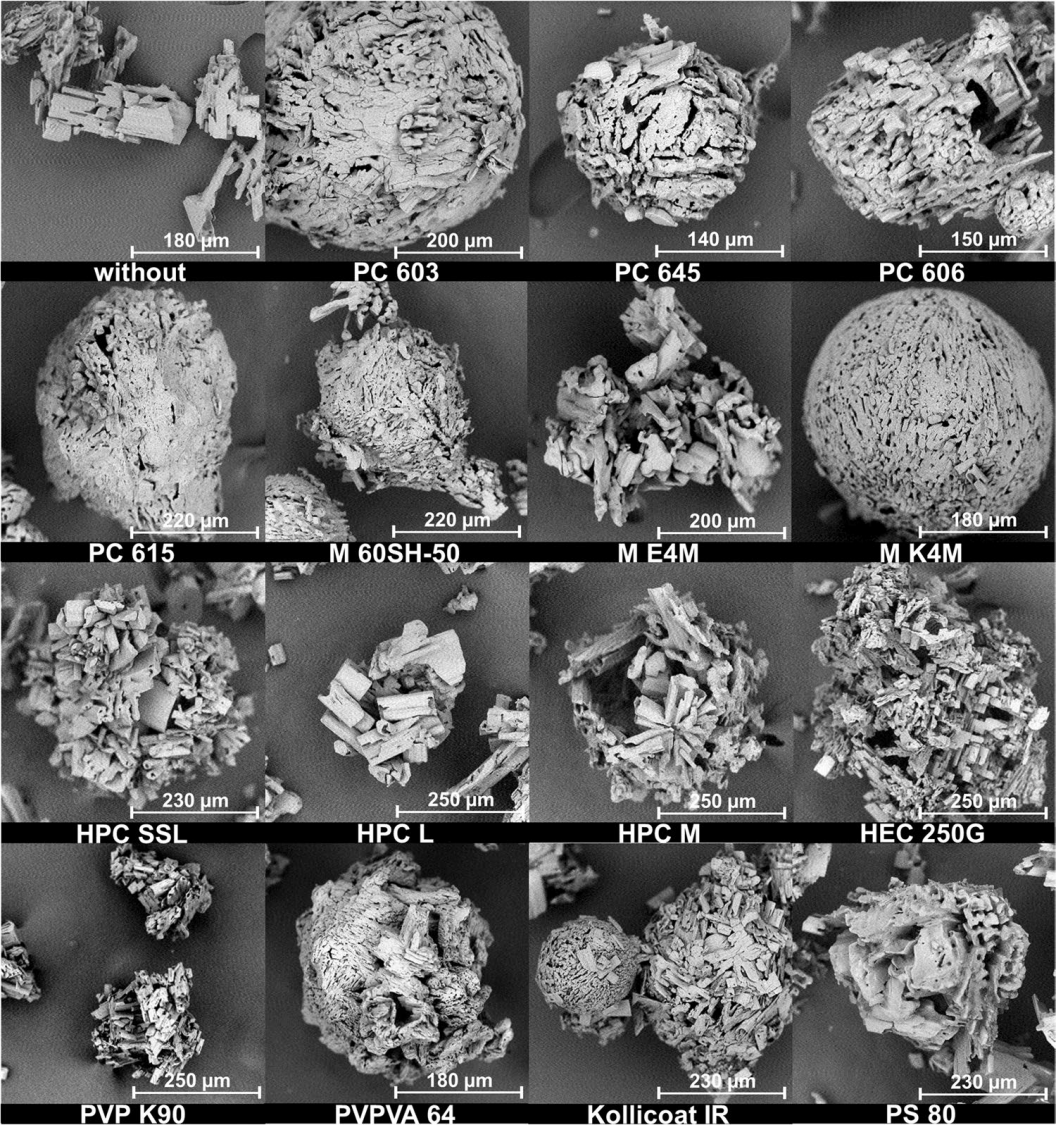
SEM images of QESD crystallized MF using different emulsion-stabilizers

SEM images (Fig. 3) showed a change in the particle morphologies with an increase in the molecular weight of the 2910 type HPMCs. The surface of the MF agglomerates became rougher and their shapes more irregular. This resulted in a reduced flowability of the agglomerates (Fig. 5). The Hausner ratio and the angle of repose both increased with decreasing amount of HPMC present in the MF solution. Measurements of the Hauser ratio showed that only the agglomerates produced with PC 603 and PC 645 still had “good” flowability according to the criteria set by the European Pharmacopoeia (28). Determining the angle of repose indicated that only the E4M HPMC would not be suitable for direct compression. As the PSDs of all of these agglomerates were similar, the reduction in flowability was therefore attributed to the rougher surface of the agglomerates. This demonstrated that the formation of emulsion droplets alone is not sufficient for the formation of spherical, good-flowing agglomerates.

It was not surprising that a reduction in the amount of polymer used led to rougher agglomerates. When the stabilizer is dissolved within the solvent phase, each droplet represents a “micro-reactor” with a limited amount of stabilizer present within. While the final aqueous solution of PC 603 contained 1.41 % (w/w) HPMC, the one containing M E4M only had 0.11 % (w/w). This means that less polymer was available for the adsorption onto the agglomerate surface and that crystal growth cannot be influenced as strongly.

When changing the HPMC substitution-type from 2910 (E4M) to 2208 (K4M), a drastic improvement in the agglomerate properties could be observed. Since both polymers have the same nominal viscosity (4000 mPa•s) and were used in similar concentrations (E4M = 0.11 % vs. K4M = 0.13 %), it was interesting to observe that the surface of the agglomerates became smoother with K4M (Fig. 3). This resulted in an improved flowability of the powder (Fig. 5), even compared to the original formulation containing PC 603. By choosing a different substitution type of HPMC, the required concentration could be reduced from 1.41 % to 0.13 %, which resembles a reduction by a factor of over 10.

**Fig. 4:**
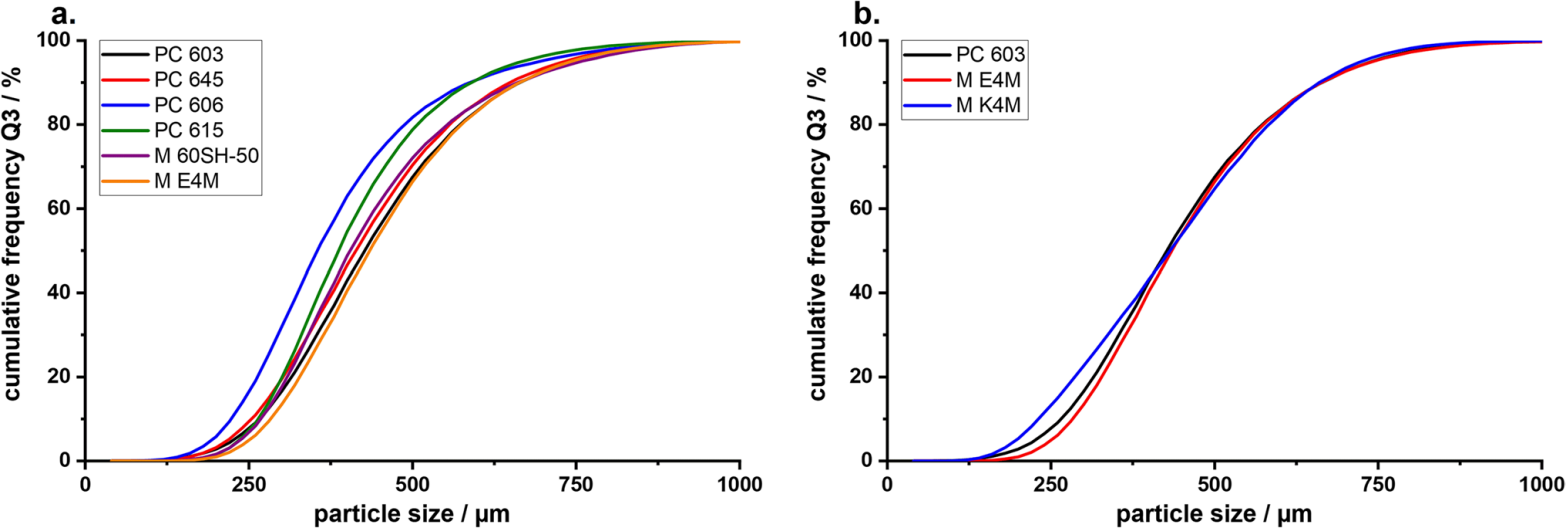
PSD of MF agglomerates crystallized in **a.** HPMC 2910 and **b.** comparison of HPMC 2910 (PC 603 and E4M) vs. 2208 (K4M), (n=1, entire batch)

A change in the PSD could not be observed (Fig. 4b), even though K4M was less surface active than E4M and PC 603. This was interesting because Wollenweber *et al*. (37) have shown that oil-in-water emulsions stabilized with HPMC 2208 had larger droplets than those with HPMC 2910. The findings indicate that the viscosity of the solution has a larger influence on the droplet size and subsequently the PSD of the produced agglomerates during a QESD crystallization than the interfacial tension. It can be hypothesized, that HPMC 2208, being more hydrophilic than the 2910-type due to the higher ratio of hydroxypropyl-groups (37), shows a higher affinity for the hydrophilic MF molecule (Log P = 0.15 (38)). Polymers are known to reduce the rate of crystal growth (39–41). As the used polymers are surface-active, they will reside not only within the droplets but also at the interface of the quasi-emulsion. An adsorption of the polymer onto the crystal faces at the interface, and the resulting reduction in the crystal growth rate in the direction of the antisolvent phase, could result in agglomerates with a smoother surface. Since the 2208 substitution-type seems to be the prominent HPMC type used in literature (14, 23–25), it would be interesting to find out whether this was a conscious decision by the authors or simply coincidence.

**Fig. 5:**
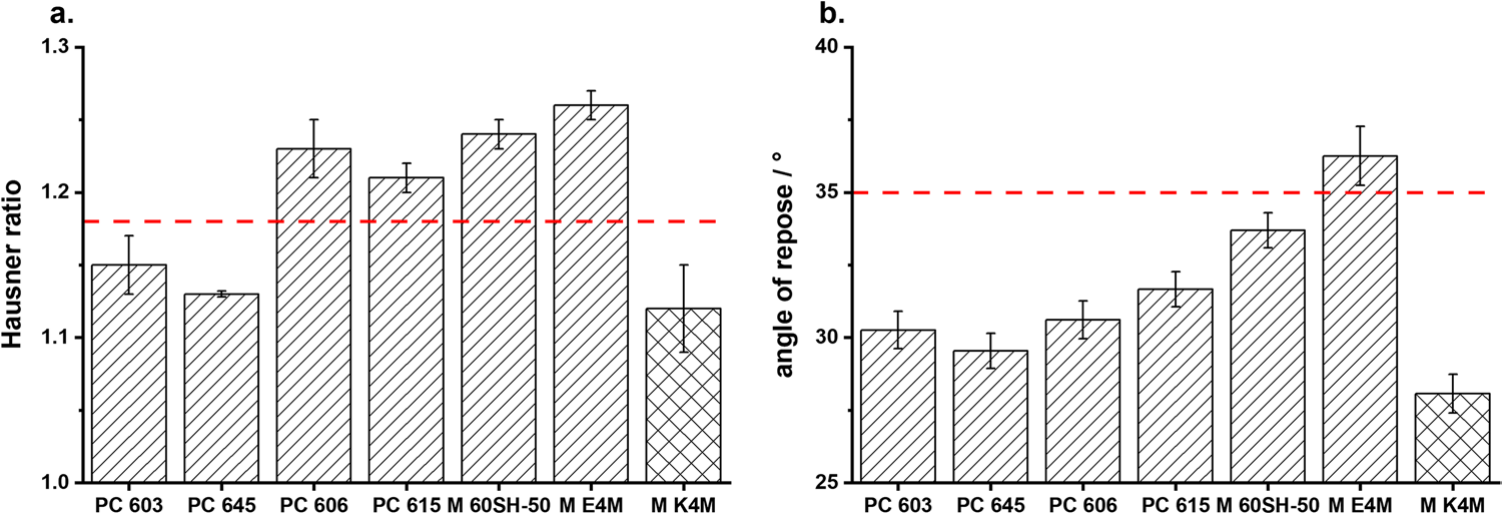
**a.** Hausner ratio and **b.** angle of repose of MF-HPMC agglomerates (diagonal pattern = HPMC 2910, crossed pattern = HPMC 2208), below red dotted line indicates “good” flowability according to European Pharmacopoeia (n=3, mean ± s)

**Fig. 6:**
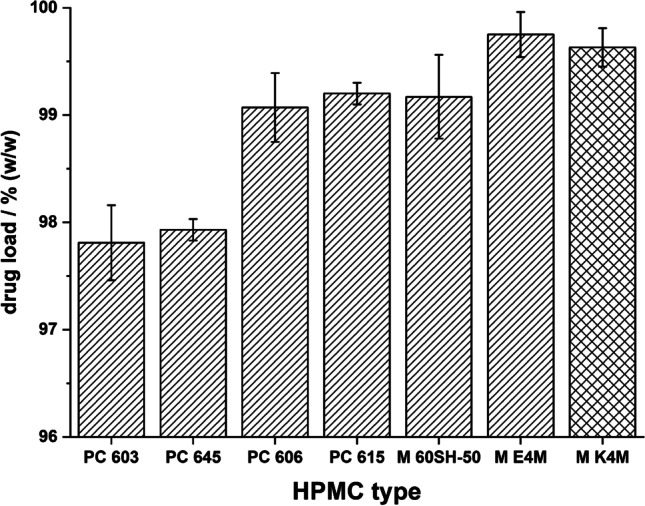
Drug load of QESD-MF agglomerates crystallized in the presence of different HPMC types (UV/Vis, λ = 233 nm, n=6, mean ± s)

**Fig. 7:**
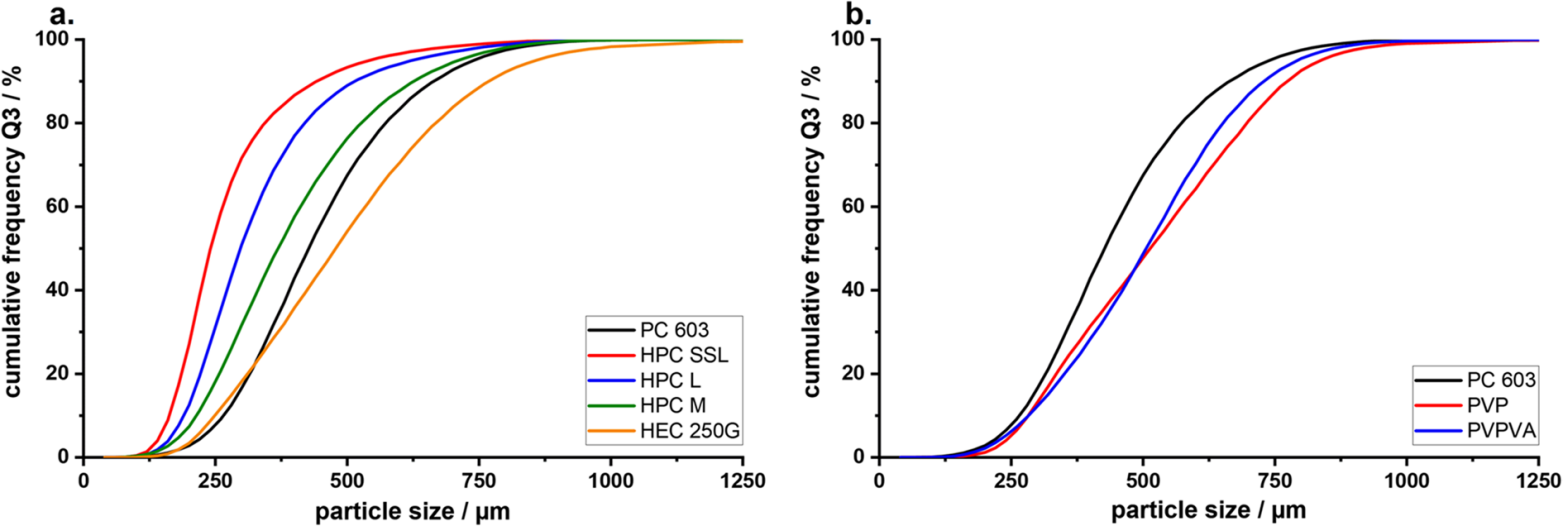
PSD of MF agglomerates crystallized in **a.** HPC and HEC and **b.** in PVP and PVPVA (n=1, entire batch)

**Fig. 8:**
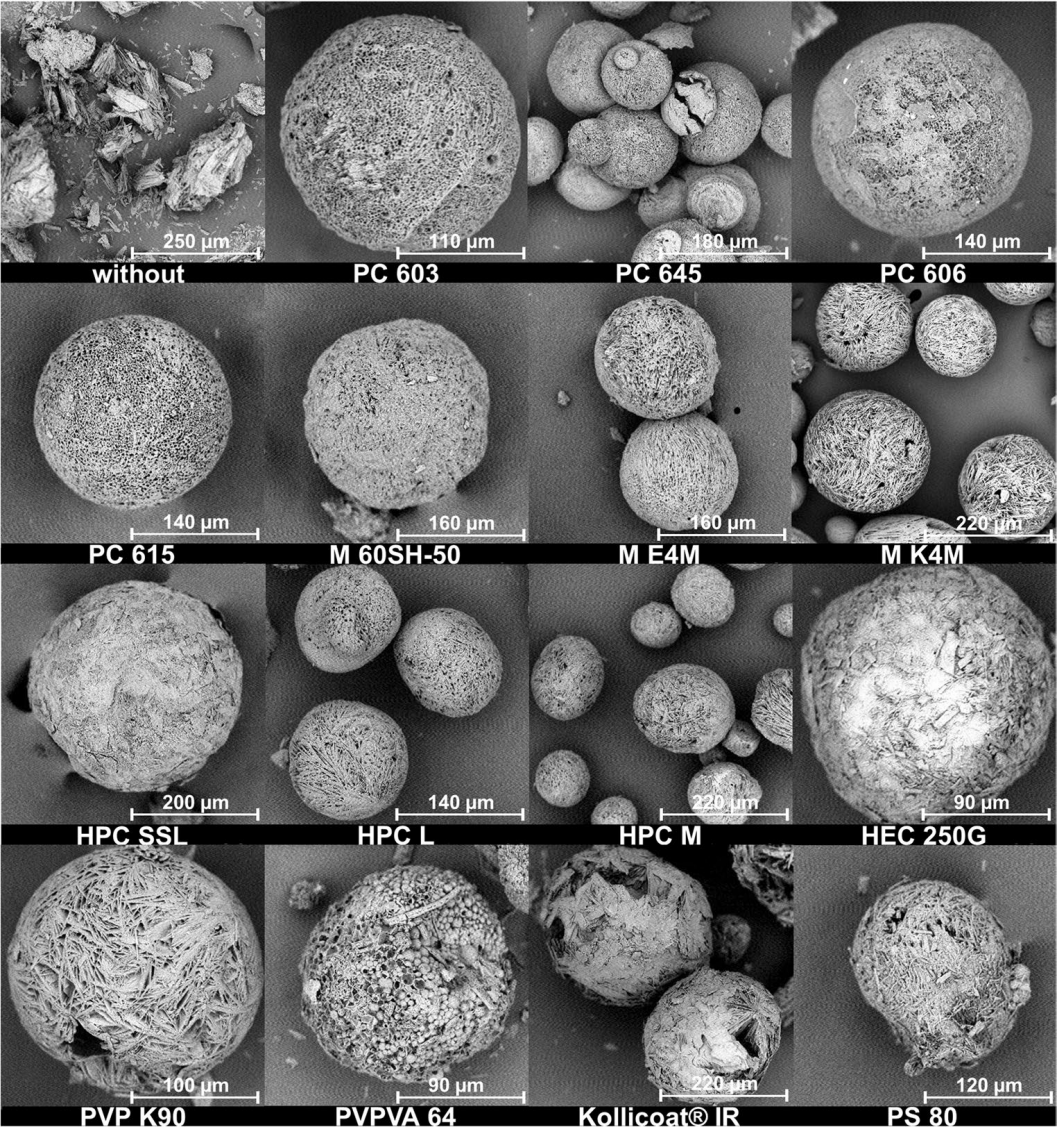
SEM images of QESD crystallized CEL using different emulsion-stabilizers

**Fig. 9:**
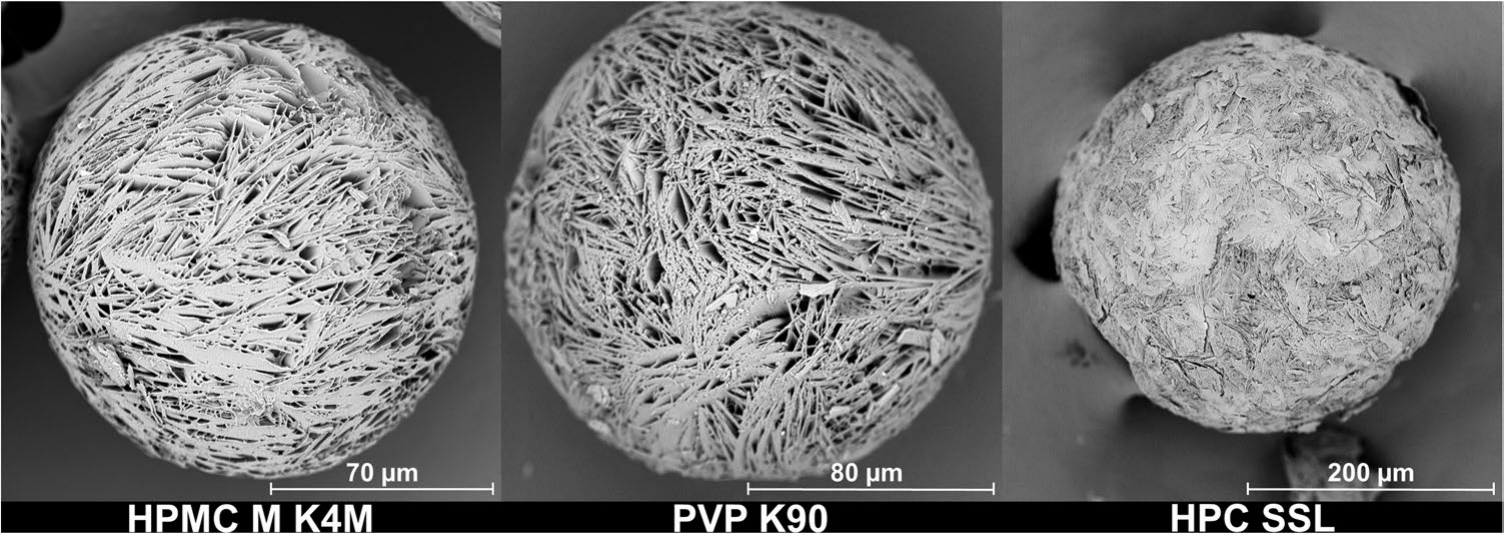
Enlarged SEM images of QESD-CEL in HPMC K4M, PVP K90 and HPC SSL

**Fig. 10:**
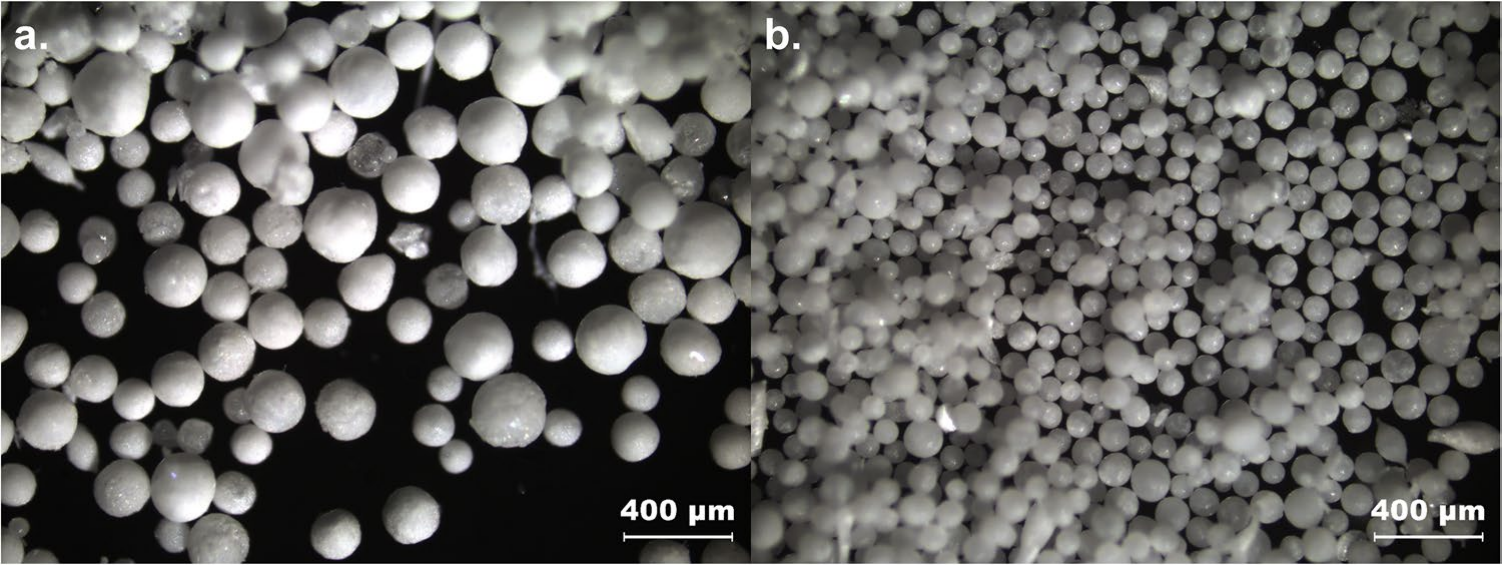
Light-microscopic images of QESD crystallized CEL in the 1000 mL crystallizer using **a.** HPMC PC 603 and **b.** HPMC M K4M as a stabilizer

**Fig. 11:**
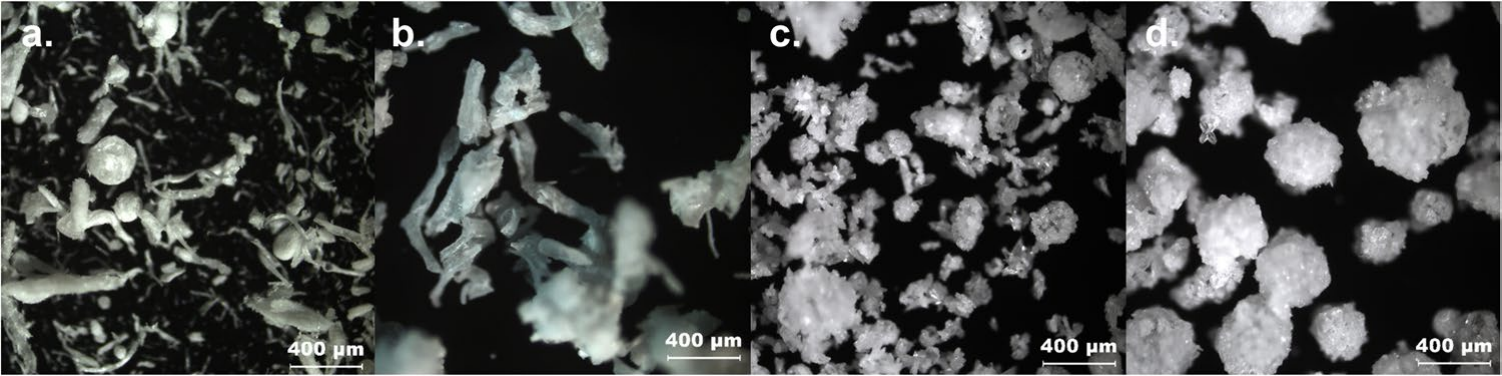
Light-microscopic images of QESD-CEL (a. and b.) and –MF (c. and d.) crystallized using 7.5 % (w/w) PVPVA as a stabilizer in **a. and c.** the outer phase and **b. and d.** the inner phase

**Fig. 12.**
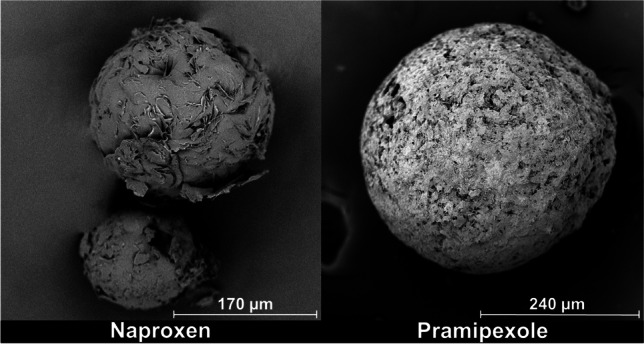
SEM images of QESD crystallized naproxen (left) and pramipexole (right) using HPMC (Pharmacoat® 603) as a stabilizer

Measuring the drug content of the MF-HPMC agglomerates showed an increased drug load of the agglomerates with a decrease in the amount of polymer used. This is in accordance with data found in literature (16) as less polymer can be adsorbed/enclosed within the MF crystals when less is available in the quasi-emulsion droplets. While the PC 603 agglomerates had a drug load of 97.81 ± 0.35 %, those stabilized with M K4M had a drug load of 99.63 ± 0.18 %. There was a linear correlation between the drug content and the HPMC concentration in solution (R=0.936, SI. 1). As HPMC can act as a binder during granulation/tableting (42), the reduction in the amount present within the agglomerates could have an influence on the tensile strength of produced tablets. This still needs to be evaluated. If an increase in the drug load of the agglomerates leads to the need for more binder present in the powder blend, little is gained.

The increase in drug load with the reduction in the polymer concentration was further confirmed by DSC measurements (SI. 2). A reduction in the enthalpy of fusion compared to reference MF was measured.

These findings show that authors need to publish the exact type of HPMC used for a QESD crystallization, ideally with the substitution type and nominal viscosity of the polymer, as this can have an impact on the agglomerate properties.

The use of HPMC Methocel™ K4M was further evaluated because 2208 type-HPMC has a higher cloud-point than the 2910-type (43). So far, the maximum concentration of the MF solution was limited by the cloud-point of PC 603, as the solution turned turbid at ~58 °C and therefore determined the amount of MF which could be dissolved. Crystallizations above the cloud-point led to irregular agglomerates and blocked tubing. Solutions of M K4M could be heated to 70 °C while remaining clear. Therefore, MF solutions with concentrations of 47.4 % (w/w) instead of 42.9 % (w/w) (11) could be used. Hopes were, that by increasing the concentration of the MF solution, the bulk density of the powder could be increased which would be advantageous for the direct compression of the material. The crystallization trials however revealed that by increasing the concentration of MF, the particle size of the agglomerates also increased (SI. 3a). While a reduction in the particle size could be achieved by increasing the stirrer rpm (SI. 3b), the bulk density of the material did not greatly increase (0.24 ± 0.01 g/mL (47.4 %, 500 rpm) vs. 0.21 ± 0.01 g/mL (42.9 %, 250 rpm)). The crust-formation within the droplet will begin at an earlier time point with an increased concentration of solute during the counter-diffusion of the solvents, as the super-saturation will be reached earlier. This leads to larger agglomerates (32). To effectively increase the bulk density of the material, the nucleation of the crystals would need to be hindered, to delay the crust-formation.

### Use of other polymers as stabilizers for QESD crystallization of MF

To evaluate the use of other surface-active polymers as stabilizers for QESD crystallizations, other cellulose-ethers (HPC and HEC), PVP, PVPVA and Kollicoat® IR were tested. HPC has been used for the spherical crystallization of naproxen (16) and mebendazole (44). The trials using the three different grades of HPC did not lead to smooth, spherical agglomerates of MF, like those with PC 603 (Fig. 3). Measuring the PSD of the MF-HPC agglomerates revealed a shift to smaller particle sizes (Fig. 7a). An increase in the molecular weight of the HPC type caused a shift to larger particle sizes, nearing those of PC 603. This was unexpected, as the dynamic viscosities of the solutions at 50 °C decreased with increasing molecular weight (Fig. 1a), which should have led to smaller emulsion droplets. As the gelation temperature decreases with increasing molecular weight (45), a further reduction in the stabilization of the quasi-emulsion was expected, which should have led to smaller particles, similar to those crystallized without a stabilizer. The MF-HPC M particles were still very rough, so that poor flowability was expected. A simple explanation for the unsuitability of HPC as a stabilizer for the QESD crystallization of MF was that by heating the MF-solution to 50 °C, the cloud-point of HPC was exceeded. This could be seen visually and was confirmed by the reduced measured dynamic viscosities.

Since the crystallization trials using HPC as a stabilizer revealed, that the polymer must be kept below its cloud-point, HEC was chosen as a further cellulose-derivative as it does not show coagulation when heated. The aqueous HEC solution did however have a higher surface tension (59.8 ± 0.9 mN/m) than PC 603 one (44.9 ± 0.1 mN/m). The dynamic viscosity of the solution at 50 °C (1.41 ± 0.05 mPa•s) was below the range of the analyzed HPMC grades (Fig. 1b). SEM images (Fig. 3) showed that the MF-HEC agglomerates did not have the desired smooth surface and were irregular in shape. The PSD measurements revealed larger agglomerates with a higher span than those produced with PC 603 (1.08 vs. 0.93). This was to be expected, as a higher interfacial tension would lead to the formation of larger droplets when the same amount of physical energy is put into the system during the emulsification. Zimmermann *et al*. (46) showed that HEC did not adsorb onto the surface of siramesine hydrochloride to the same extent as HPMC or HPC. The increased hydrophilicity of HEC and the resulting decreased interaction with MF could lead to irregular agglomerates as crystal growth cannot be sufficiently hindered at the interface (Fig. 6).

Kollidon® 90 F (PVP), Kollidon® VA 64 (PVPVA) and Kollicoat® IR were selected as further surface-active polymers which did not show coagulation when heated to 50 °C. The solutions showed a slightly decreased dynamic viscosity at 50 °C compared to the PC 603 solution. In the concentrations used (PVP = 1.25 %, VA 64 = 7.50 %), the PVP solution had a higher surface tension than the PVPVA one. Both polymers showed in increase in the PSD compared to PC 603 (Fig. 7b). PVPVA is more hydrophobic than PVP due to the additional vinyl acetate groups present within the N-vinylpyrollidone chain. The increased hydrophobicity of PVPVA might explain why the MF-PVPVA agglomerates were more spherical in shape (Fig. 3) and had a smoother surface than those of PVP. Ilevbare *et al*. (34) showed that PVPVA was more effective than PVP at reducing the crystal growth rate of different structurally diverse compounds. The adsorption of the crystal faces at the interface could therefore lead to the formation of smoother agglomerates. The authors would however not recommend PVPVA as a stabilizer, as the high amount of polymer present within the system lead to a gel-like cake after the first filtration, which was difficult to wash. Kollicoat® IR was also not a suitable stabilizer for the QESD crystallization as the agglomerates were more irregular in shape than those produced with PC 603 (Fig. 3) and the high polymer concentration (5 %) led to a gel-like cake after filtration.

Polysorbate 80, in a concentration of 0.008 % (w/w) was chosen as a surfactant, as it only reduced the surface tension of the aqueous solution to that of the PC 603 one, without changing its viscosity compared to water at 50 °C (0.64 ± 0.04 mPa•s vs. 0.66 ± 0.02 mPa•s for water). SEM images (Fig. 3) showed, that PS 80 was not able to sufficiently stabilize the emulsion, as irregularly formed agglomerated were obtained. This confirmed the idea, that the viscosity of the solution has a higher impact on the size and stability of transient droplets than the reduction of the interfacial tension.

Ranking the polymers by their hydrophobicity (HPMC 2910 > HPMC 2208 > PVPVA > PVP) (34, 43) shows, that in the case of MF, a certain hydrophobicity for adequate adsorption of the polymer on the crystal surface is required, so that smooth and spherical particles can be generated.

### QESD crystallization of celecoxib

CEL was selected as a poorly water-soluble API, as the QESD crystallization system would therefore have to be inversed; the API is dissolved in acetone and the stabilizer is dissolved in the antisolvent, water. As the authors only had a limited availability of the material, the experiments had to be conducted on a smaller scale and the agglomerates were only characterized using microscopic images. However, to verify however that the described CEL-QESD crystallizations could be performed at a larger scale, two crystallizations of CEL were performed using PC 603 and M K4M as stabilizers using the setup and respective volumes and pump-rate used of the MF experiments.

As QESD crystallizations have already been described for CEL (14, 26), the authors wanted to analyze whether the findings of those publications could be verified by these experiments. Chen *et al*. reported that CEL could be spherically crystallized by using HPMC as a stabilizer, but not HPC or PVP. The authors explained this phenomenon by analyzing the interaction between CEL and HPMC, HPC and PVP using peak-shifts of ^1^H-NMRs. The interaction between the polymers HPC and PVP and CEL was reduced compared to HPMC and CEL, which led the authors to the conclusion that HPC and PVP cannot sufficiently stabilize the emulsion. This was confirmed by microscopic images of the emulsion. However, the authors used the polymers in a set concentration of 0.1 % (w/w), which leads to solutions of different viscosities. An adjustment of the viscosities might therefore lead to different results.

Crystallizations of CEL with the polymers at the concentrations described in Table II led to the formation of spherical particles in all cases (Fig. 8). However, especially in the case of PVPVA and Kollicoat® IR, alongside the spherical agglomerates, fibrous particles (like those seen in Fig. 11a) were found which the authors assume to be polymer which precipitated during the crystallization. Another explanation could be related to the setup using a simple magnetic stirrer, which could have led to an inhomogeneous formation of the emulsion droplets. The yield of spherical particles for PVPVA and Kollicoat® IR was estimated to be ~30%. The authors would therefore not recommend these polymers for QESD. Furthermore, the high amount of polymer required to achieved the desired dynamic viscosity (7.5 % for PVPVA and 5 % for Kollicoat® IR) of these stabilizers led to a gel-like sediment during filtration which was difficult to wash. Even PS 80 led to the formation of agglomerates, however these were not as spherical or smooth as those produced with HPMC. The authors would not advise the use of non-ionic surfactants as these can lead to tablets with a reduced strength due to their lubricating effect (13).

The findings of Chen *et al*. (14), that the specific interaction between the API and polymer is essential for the formation of spherical particles could not be confirmed as HPMC, HPC and PVP could all be used to stabilize the transient emulsion. This demonstrates the impact of the viscosity of the solutions on the agglomerate morphology: Chen *et al*. used 0.1 % (w/w) solutions of the polymers as an antisolvent, even though the nominal viscosity of the polymers used are very different (47–49). As Maghsoodi *et al*. (16) have shown, the viscosity of the solution has a significant impact on the agglomerate properties. This is why the authors believe, that a screening method via the described viscosity method could be a superior one.

Since all 14 polymers analyzed in this screening could be used to generate spherical particles of CEL, it could be shown that the stabilization of the transient emulsion during a QESD crystallization can be achieved by increasing the viscosity of the antisolvent phase. However, the SEM images showed that the type of polymer used can have a definite impact on the morphology of the CEL particles. While those crystallized in the presence of HPMC and HPC were mainly spherical and smooth, those crystallized in the presence of HEC, PVPVA and Kollicoat® IR showed an irregular surface (Fig. 8). This could be due to differences in the interaction between CEL and the respective polymer (34). The hypothesis could be formed, that in general, the stabilization of the emulsion leads to spherical particles, while the specific polymer-API interaction leads to the formations of different crystal forms and structures of the primary particles within the agglomerates. The QESD-CEL agglomerates produced with M K4M and PVP showed a needle-like morphology of the primary particles, while those stabilized with HPC SSL had a plate-like morphology (Fig. 9).

Comparing the results of the QESD MF and CEL crystallizations, the largest difference between the two is the absolute amount of polymer present during crystallization. When the polymer is present within the inner phase (MF), each emulsion droplet has a limited amount of stabilizer present within. For the QESD crystallization to be successful, this stabilizer has to 1. remain dissolved at the required temperature, 2. be able to sufficiently stabilize the emulsion droplets to allow for a steady counter-diffusion of solvent and antisolvent and 3. be able to modify the crystal growth rate at the interface, so that particles with a smooth surface can be generated. When the stabilizer is dissolved in the outer phase (CEL), it is abundantly present. There is a lot more polymer present which can adsorb onto the crystals at the interface and thereby modify the crystal growth rates. This can compensate for a reduced affinity to the crystal surface, which cannot be done if the stabilizer is only present within the droplets. Therefore, systems where the stabilizer is present within the antisolvent seem to be more robust.

To verify, that the crystallizations of CEL can also be performed on a larger scale, two QESD crystallizations were performed where the quasi-emulsion was stabilized using HPMC PC 603 and M K4M. Figure 10 shows, that spherical particles could be produced in both cases and the powder showed a good flowability when shaken by hand.

### Effect of the location of the stabilizer

As stated previously, one of the differences between the MF and CEL systems analyzed, was the location of the stabilizer: for the MF crystallizations they were present within the solvent phase, for the CEL ones within the antisolvent. Therefore, the absolute amount of polymer available for the crystallization was around 17-times higher for the CEL trials. An experiment was therefore conducted where the stabilizer was either dissolved in the solvent or the antisolvent and the resulting particle morphology was evaluated. PVPVA, in the concentration of 7.5 % (w/w), was used as a stabilizer as it was the only polymer used in the screening which was soluble in both acetone and water. Looking at the QESD-CEL particles (Fig. 11a and b), it is apparent that while some spherical particles can be generated when PVPVA was present in the outer phase (antisolvent), none could be found when it was dissolved within the solvent phase. Microscopic images of the QESD-MF particles (Fig. 11c and d) revealed more spherical and uniformly shaped particles when PVPVA was dissolved in the solvent phase. This is in contrary to the hypothesis stated previously, however an observation made during the crystallization with PVPVA in the outer phase could explain this phenomenon. It could be seen that the droplets of the MF solution were not finely dispersed as in the previous trials, but instead web-like strings were formed (see SI. 4). The high amount of polymer present in the antisolvent may have resulted in a high adsorption onto the MF crystals, so that the formation of fine droplets and thereby spherical particles was hindered. UV-Vis measurements of the two different MF-PVPVA agglomerates showed a significant increased drug-load when PVPVA was present in the solvent phase (98.12 ± 0.23 %) compared to the antisolvent one (95.88 ± 0.29 %, *p*-value < 0.00001).

### Suitability of Pharmacoat® 603 as a stabilizer for the QESD crystallization of other drugs

As many APIs using various stabilizers have been successfully crystallized by different working groups using the QESD method, the authors wanted to see whether the current system using Pharmacoat® 603 HPMC could be used for other APIs. For this, a screening of both poorly and highly water-soluble APIs available to the authors was done. QESD crystallizations were performed using the apparatus described for the MF crystallizations, using the same volumes of the solutions and pump-rates.

Pramipexole (PX) was chosen, as it had a high solubility in water and a low solubility in acetone. A QESD crystallization method for PX has not been published in literature. SEM images (Fig. 12) showed that spherical agglomerates of PX were successfully produced. Manual agitation of the powder also showed a good flowability and a Hausner ratio of 1.10 ± 0.02 was determined. Selegilin hydrochloride, metoprolol tartrate and salbutamol sulfate (19) were also evaluated. However, the first two did not precipitate and remained soluble in the antisolvent phase and salbutamol sulfate produced a viscous gel which could not be filtered.

QESD crystallizations of the poorly water-soluble drug naproxen (NP) have already been described in literature (16, 21), using HPC as a stabilizer. The concentration of the NP solution in acetone was the same as described by Maghsoodi *et al*. (16). The scale of the experiment was however larger (100 mL vs. 600 mL antisolvent). SEM images (Fig. 12) revealed, that PC 603 could also be used as a stabilizer to spherically crystallize NP. The authors would however recommend to use a HPMC-type with a higher molecular weight, and therefore requiring a lower concentration, as the spherical agglomerates were trapped within a gel-like paste which had to be washed extensively to obtain a free-flowing powder. This washing process also led to a reduction in yield.

It has to be said that the authors do not believe that aqueous solution of PC 603 with a dynamic viscosity of 3.25 mPa•s is the ideal stabilizer for QESD crystallizations as other APIs may interact differently with the polymer, have different nucleation- and crystal-growth-rates and different solubilities. However, once a suitable polymer has been found in a certain concentration, others can be evaluated with solutions of similar viscosities. Other polymers might be more suitable as they can be cheaper, influence the micromeritic properties of the agglomerates or improve dissolution.

## Conclusion

Quasi-emulsion solvent-diffusion crystallizations require the formation of transient emulsion droplets of a drug or excipient solution within a suitable antisolvent. The exact role of the emulsion stabilizers used is however still unclear, as both the simple stabilization of the emulsion droplets as well as specific interactions between the polymer and drug are discussed in literature. This work has shown that QESD systems behave very differently when the surfactant is dissolved in the solvent or antisolvent. When the dissolution of the stabilizer is possible in the antisolvent, the QESD systems seem to be more robust, as a higher amount of polymer is available to the system. Crystal growth at the interface can therefore be hindered more effectively, which leads to the formation of smooth agglomerates. While metformin hydrochloride could only be spherically crystallized using 3 types of HPMC, QESD celecoxib crystallizations could be stabilized with a variety of polymers. The viscosity of the solutions has to be kept within bounds, since this can have an impact on the morphology of the agglomerates. This is especially important during the screening of polymeric surfactants during the development during a QESD crystallization process.

## Supplementary Information

ESM 1 (DOCX 236 kb)
