# Supplementary material for: Towards a better understanding of the role of stabilizers in QESD crystallizations
Source: Pharm Res. 2022 Mar 9;39(12):3123–36. doi: 10.1007/s11095-022-03212-2 (PMC9780136; doi:10.1007/s11095-022-03212-2)
Supplement: Supplementary file 1 — (DOCX 236 kb) [file 11095_2022_3212_MOESM1_ESM.docx]

**SUPPORTING INFORMATION**

**SI 1.** Linear fit of the drug load of HPMC-MF agglomerates vs. the concentration of HPMC in the MF solution (n=6, mean ± s)

|  | **Peak / °C** | **Enthalpy of fusion / J/g** |
| --- | --- | --- |
| **Reference** | 232.34 | 322.61 |
|  | 232.19 | 321.52 |
| **PC 603** | 230.03 | 286.94 |
|  | 229.91 | 281.60 |
| **PC 645** | 230.37 | 287.13 |
|  | 230.28 | 291.89 |
| **PC 606** | 230.51 | 295.39 |
|  | 230.63 | 300.21 |
| **PC 615** | 230.62 | 306.50 |
|  | 230.23 | 300.72 |
| **M 60SH-50** | 230.44 | 283.93 |
|  | 230.73 | 314.39 |
| **M E4M** | 230.12 | 301.75 |
|  | 230.41 | 286.44 |
| **M K4M** | 230.32 | 304.95 |
|  | 229.97 | 306.90 |

**SI 2.** DSC measurements of MF-HPMC agglomerates

**SI 3.** PSDs of MF-QESD crystallization using 0.25 % HPMC M K4M as a stabilizer, **a.** influence of MF concentration (%, w/w) and **b.** influence of stirrer rpm (n=1, entire batch)


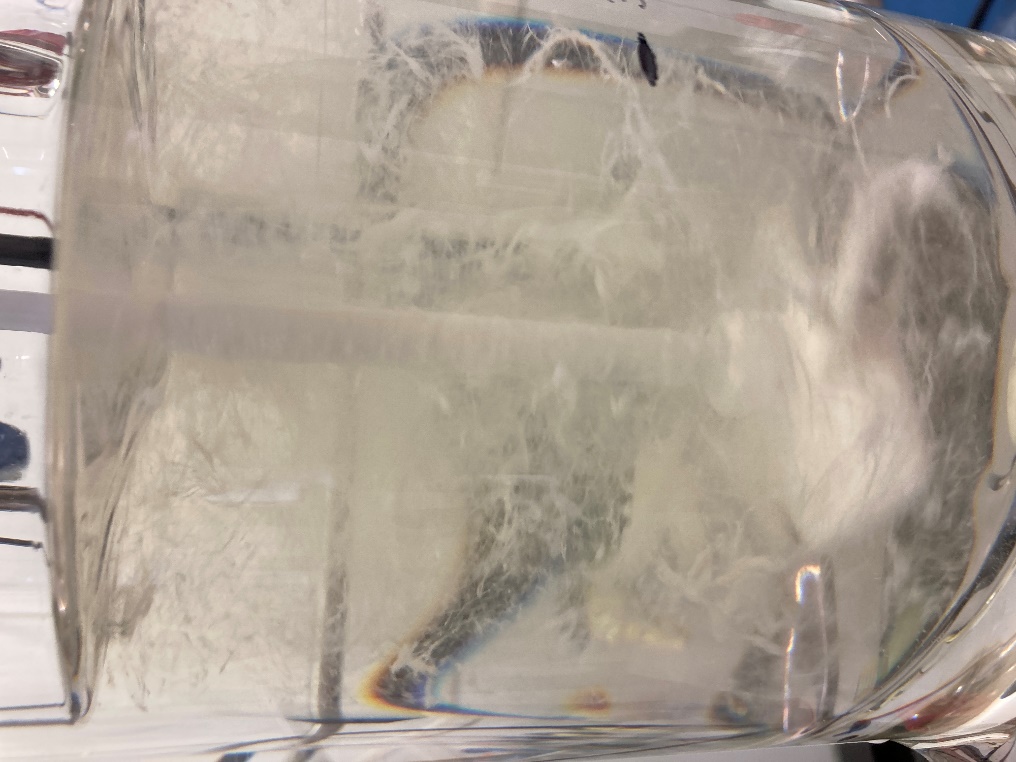


**SI 4.** QESD-MF crystallization with 7.5 % (w/w) PVPVA in the antisolvent phase
